# Cystoscopic submucosal indocyanine green marking in robot-assisted radical nephroureterectomy: a multicenter cohort study

**DOI:** 10.3389/fonc.2026.1957208

**Published:** 2026-09-28

**Authors:** Runyi Wang, Menghua Wu, Haozhou Wang, Hui Liu, Xin Zheng

**Affiliations:** 1 Department of Urology, Beijing Chao-Yang Hospital, Capital Medical University, Beijing, China; 2 Department of Urology, Beijing Hospital of Traditional Chinese Medicine, Capital Medical University, Beijing, China; 3 Department of Urology, Beijing Daxing District Hospital of Integrated Chinese and Western Medicine, Beijing, China

**Keywords:** fluorescence-guided surgery, indocyanine green, radical nephroureterectomy, robot-assisted surgery, upper tract urothelial carcinoma

## Abstract

**Background:**

Distal ureter and bladder cuff dissection can be difficult during robot-assisted radical nephroureterectomy (RA-RNU) for upper tract urothelial carcinoma (UTUC), particularly in the deep pelvis where tactile feedback is limited. We evaluated cystoscopic submucosal indocyanine green (ICG) marking around the ureteral orifice as a navigation aid for this step.

**Methods:**

Adults who underwent RA-RNU with formal bladder cuff excision for clinically localized UTUC (≤T3, M0) at three centers between January 2019 and November 2024 were retrospectively analyzed. Outcomes were compared between submucosal ICG-guided and conventional non-ICG procedures. The primary endpoint was video-derived distal ureter and bladder cuff identification time; postoperative care-process measures were descriptive, and other perioperative and oncological outcomes were exploratory.

**Results:**

The analysis included 82 patients (43 ICG, 39 non-ICG). The ICG group was older, and the largest baseline imbalances by SMD involved age and pathological T stage. Median identification time was 9.0 min with ICG and 19.3 min without ICG (p < 0.001). In the primary parsimonious model, the geometric mean ratio was 0.49 (HC3 95% CI 0.45–0.53; p < 0.001). No statistically significant between-group difference was observed in Clavien–Dindo grade. During exploratory follow-up, intravesical recurrence occurred in 1/43 versus 2/39 patients and extravesical recurrence in 3/43 versus 8/39; event counts were insufficient for comparative inference.

**Conclusions:**

Submucosal ICG marking around the ureteral orifice was associated with faster distal ureter and bladder cuff identification during RA-RNU. Prospective studies are needed to confirm reproducibility and evaluate injection-specific safety and longer-term oncological outcomes.

## 1 Introduction

Upper tract urothelial carcinoma (UTUC) represents 5–10% of urothelial malignancies, and population-based studies indicate that its incidence has increased in recent decades (1, 2). For high-risk localized disease, radical nephroureterectomy (RNU) requires removal of the kidney, the full ureter, and a bladder cuff (3–5). Robot-assisted RNU (RA-RNU) offers a minimally invasive route to the same operation, with reported oncological outcomes broadly comparable to open surgery in selected patients (6, 7). The distal ureter and bladder cuff nevertheless remain technically demanding (8, 9). In the deep pelvis, limited tactile feedback makes the surgeon increasingly dependent on visual anatomy (10, 11).

Near-infrared imaging with indocyanine green (ICG) can improve intraoperative visualization in robotic urology (12–14). Its usefulness for ureteral localization depends on the distribution of the dye. Intravenous administration may produce vascular background fluorescence because ICG binds rapidly to plasma proteins (15, 16). Retrograde intraluminal or transurethral delivery can mark the distal ureter directly but requires additional urinary tract instrumentation in patients with UTUC (17). In UTUC, the oncological relevance of additional urinary tract manipulation remains uncertain. Two retrospective studies associated diagnostic ureteroscopy or retrograde upper-tract manipulation with subsequent intravesical recurrence (18, 19).

We used cystoscopic submucosal injection around the ipsilateral ureteral orifice to create a fluorescence mark at the ureterovesical junction without filling the urinary lumen. ICG has favorable near-infrared properties, strong protein binding, and a low reported incidence of adverse reactions (15, 20). Local submucosal placement was expected to provide a stable visual cue during pelvic dissection while limiting injection pressure and systemic background signal.

We evaluated this technique in a multicenter retrospective cohort of patients undergoing RA-RNU, with distal ureter and bladder cuff identification time as the primary endpoint. Eligibility, treatment classification, and follow-up were defined consistently across groups, and oncological outcomes were analyzed on an exploratory basis.

## 2 Methods

### 2.1 Study design

We conducted a three-center retrospective cohort study comparing RA-RNU with cystoscopic submucosal ICG marking and conventional RA-RNU without fluorescence marking. The institutional review board at each center approved the protocol and waived informed consent for analysis of anonymized retrospective data.

The date of RA-RNU served as the common index date (T_0_). Eligibility, treatment classification, outcome ascertainment, and follow-up were aligned to this date. Patients were classified according to the intraoperative strategy actually used at the index operation. Distal ureter and bladder cuff identification time was the primary endpoint; **Supplementary Table 1** summarizes the study design and operational definitions.

### 2.2 Study population

The source cohort comprised adults who underwent RA-RNU with formal bladder cuff excision for UTUC from January 2019 through November 2024. Eligibility required pathological confirmation of urothelial carcinoma, nonmetastatic disease up to clinical T3, completion of RA-RNU with bladder cuff excision, and sufficient records for the primary perioperative endpoint and follow-up status. Eligibility was assessed at T_0_.

Exclusion criteria were cT4 or M1 disease, non-urothelial histology, synchronous bladder cancer requiring separate treatment at surgery, previous radical cystectomy, tumor involving the distal or intramural ureter or ureterovesical junction, a nonrobotic procedure, insufficient clinicopathological or follow-up information, or another fluorescence-localization strategy such as retrograde high-pressure intraluminal ICG instillation. **Figure 1** shows cohort assembly.

**Figure 1:**
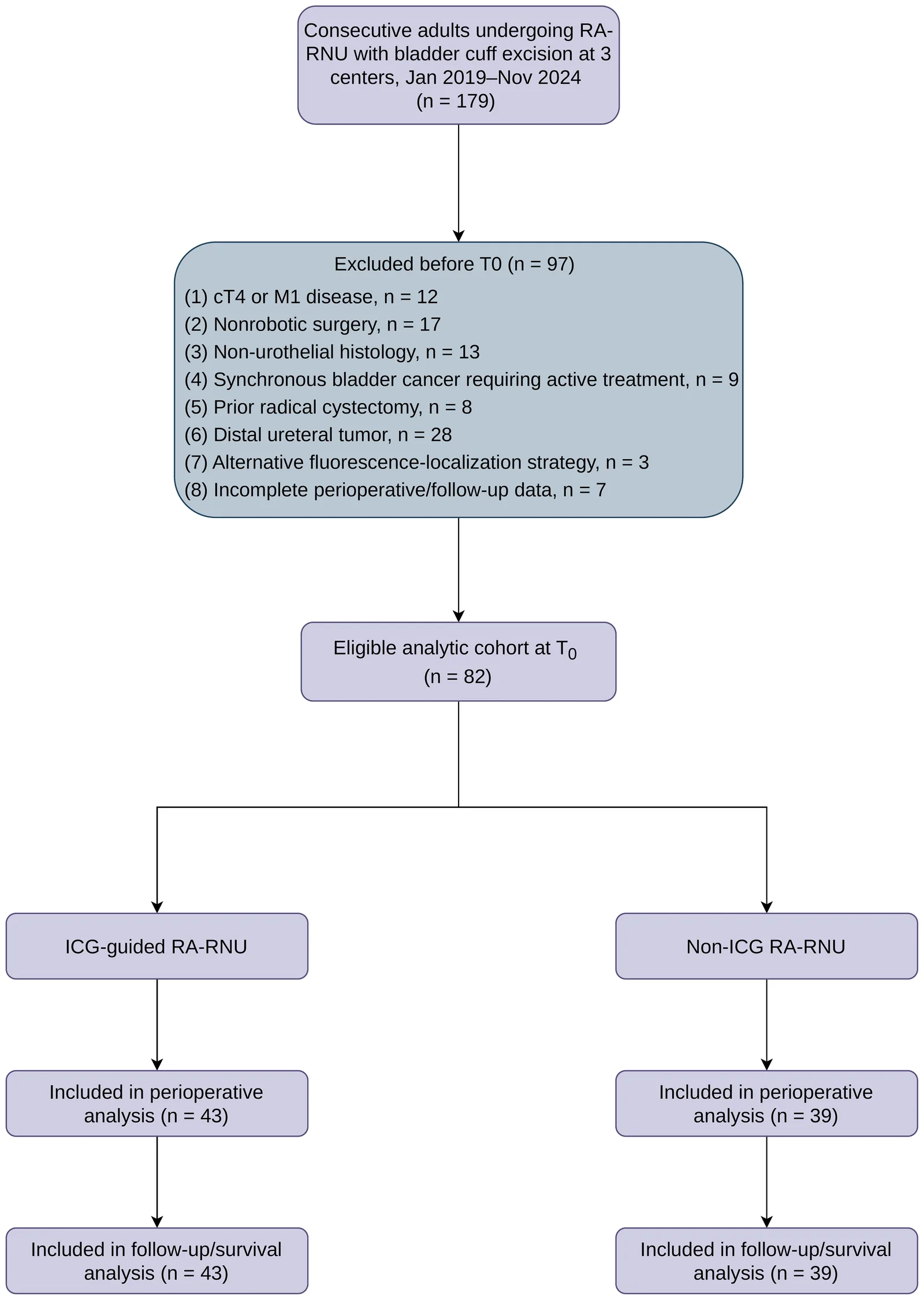
Study flow diagram. Patient screening and construction of the analytic cohort. Abbreviations: ICG, indocyanine green; non-ICG, without indocyanine green guidance; RA-RNU, robot-assisted radical nephroureterectomy.

### 2.3 Surgical technique

#### 2.3.1 Patient positioning and submucosal ICG injection

After induction of general anesthesia, patients were placed in a modified flank position with the legs supported in a split configuration. This setup maintained urethral and abdominal access throughout the case and allowed the cystoscopic and robotic phases to proceed without repositioning or re-draping.

In ICG-guided procedures, cystoscopy was performed after positioning and before robotic docking. ICG was prepared at a concentration of 2.5 mg/mL by dissolving 25 mg in 10 mL of sterile water. This preparation concentration was selected based on previously reported urological fluorescence practice, including a retrograde ureteral-localization technique using the same preparation (17). However, that technique involved intraluminal administration and does not establish an optimal concentration or dose for bladder-submucosal injection. Under direct vision, a 4–6 Fr injection needle was advanced into the trigonal bladder submucosa. Small aliquots were injected slowly at 4–6 sites around the ipsilateral ureteral orifice until discrete localized submucosal blebs were formed; the unused solution was discarded (**Figure 2**). Each injection was delivered at low pressure, with care taken to avoid the ureteral lumen and intravascular injection. A urinary catheter was inserted after injection and clamped for approximately 5 min to permit local tissue uptake; drainage was then restored, and the bladder remained decompressed during the subsequent robotic procedure.

**Figure 2:**
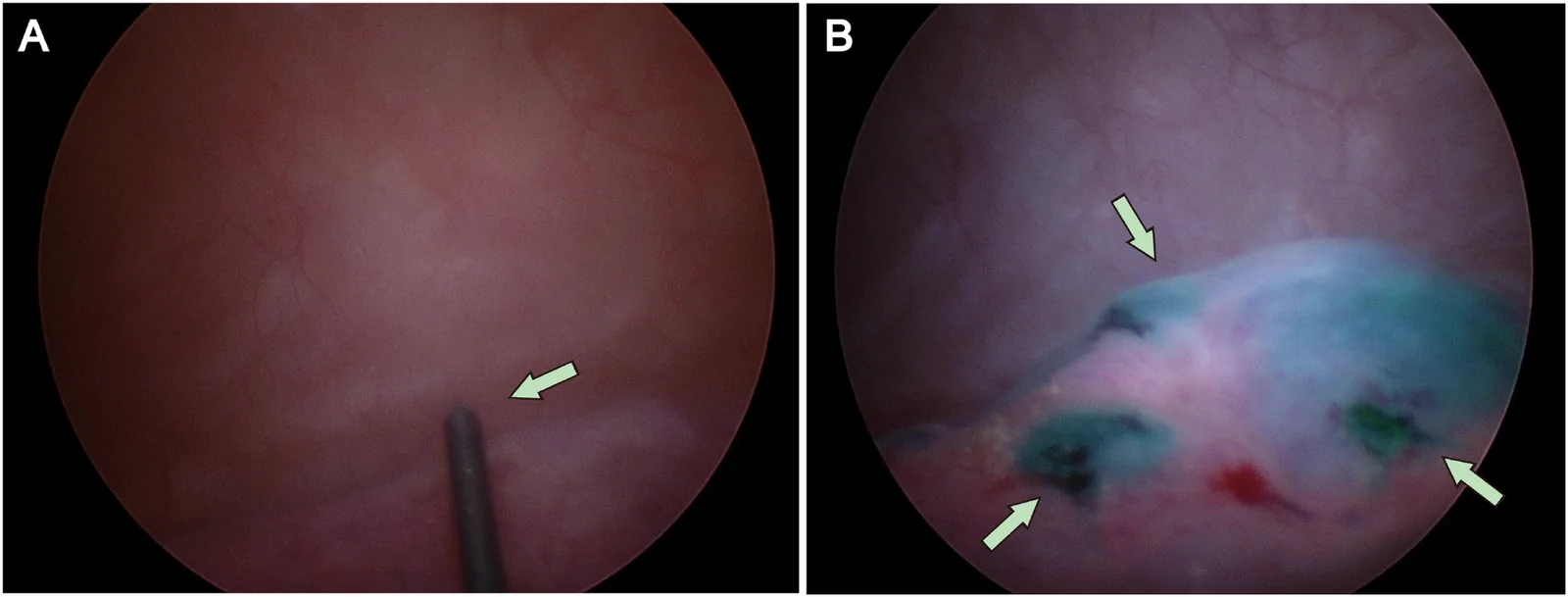
Cystoscopic submucosal indocyanine green marking around the ipsilateral ureteral orifice. **(A)** Cystoscopic view of the ipsilateral ureteral orifice before injection. **(B)** Circumferential submucosal deposits at 4–6 sites around the ipsilateral ureteral orifice, showing immediate localized mucosal elevation after injection. Arrows indicate the orifice and injection sites.

Patients in the non-ICG group underwent RA-RNU with bladder cuff excision according to the usual robotic approach at each institution, without fluorescence localization.

All procedures were performed or directly supervised by two senior robotic urologists. The key operative steps were standardized across centers. Before the study period, each surgeon had completed ≥ 50 UTUC RNU/RA-RNU procedures and more than 300 robotic urologic operations.

#### 2.3.2 Single-position nephroureterectomy and fluorescence-guided cuff excision

After docking, the renal hilum was controlled and the renal vessels were divided. The ureter was then identified near the lower pole of the kidney and clipped distal to the tumor before further ureteral mobilization. Dissection proceeded caudally along the ureter, with a second clip placed on the distal ureter before ureterovesical-junction dissection and bladder cuff excision.

When dissection reached the pelvis, the platform’s near-infrared mode was activated in ICG-guided cases. The submucosal fluorescence mark delineated the ureterovesical junction and intramural ureter against adjacent pelvic fat and detrusor muscle (**Figure 3**).

**Figure 3:**
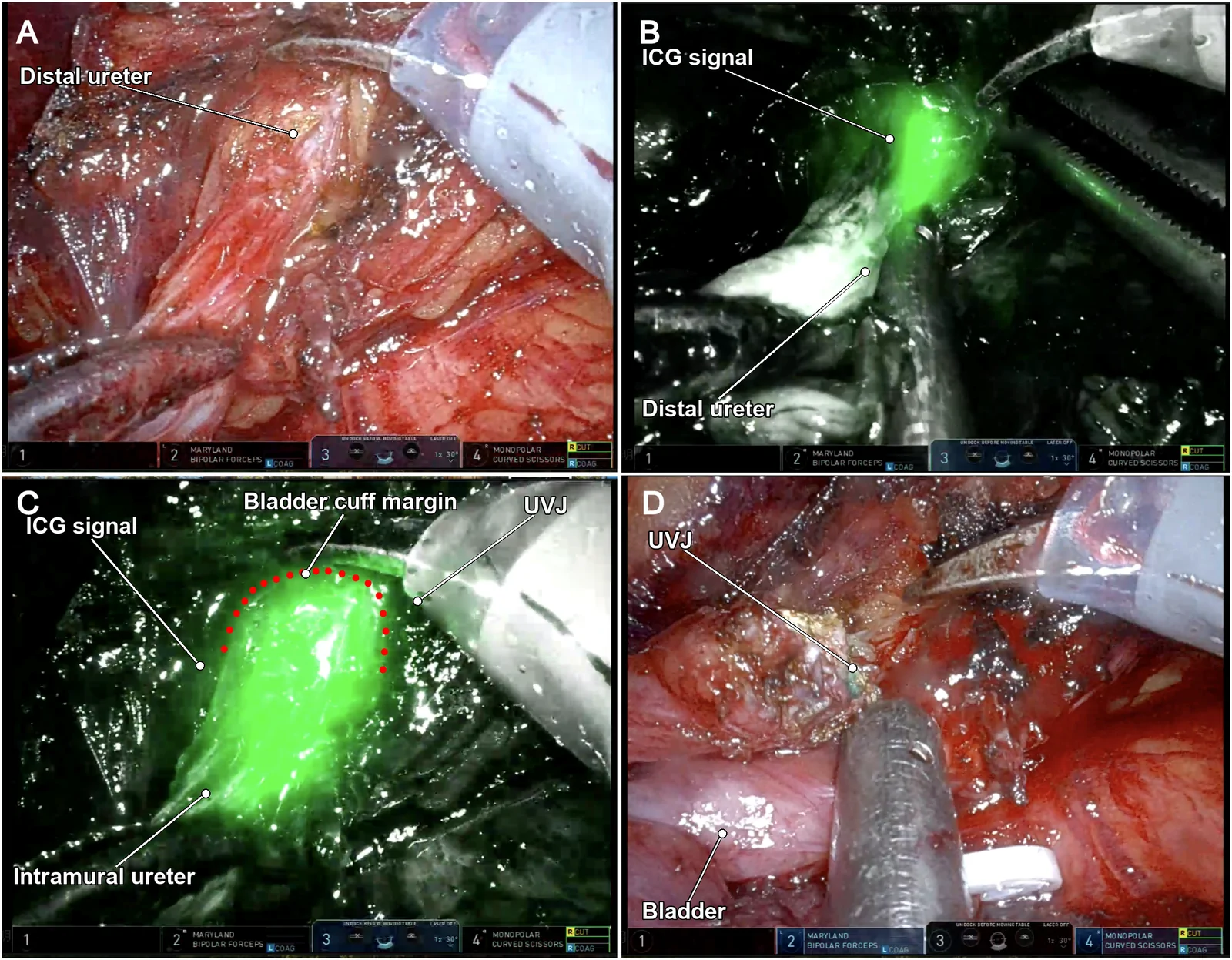
Fluorescence-guided identification of the distal ureter and bladder cuff during robot-assisted radical nephroureterectomy **(A)** White-light view of pelvic dissection showing the distal ureter. **(B)** Near-infrared view showing the submucosal ICG signal at the ureterovesical junction, with the distal ureter coursing toward the fluorescent landmark. **(C)** The fluorescence mark delineates the ureterovesical junction and the intramural ureter and guides the planned bladder cuff margin (red dotted line). **(D)** White-light view during circumferential bladder cuff excision at the level of the ureterovesical junction. ICG, indocyanine green; UVJ, ureterovesical junction.

The fluorescence mark oriented circumferential bladder cuff incision. The kidney, ureter, and intact bladder cuff were removed en bloc, and the bladder defect was closed with a continuous watertight suture. Both groups followed the same oncological principle of complete en bloc removal. Postoperatively, all patients received a single intravesical instillation of pirarubicin within 24 h after surgery. Pirarubicin 50 mg was diluted in 50 mL of 5% glucose–sodium chloride solution and retained in the bladder for 30 min, with patient repositioning every 10 min. Pelvic drains were generally removed when output was <100 mL/day and there was no evidence of bleeding, urinary leakage, or infection. Urethral catheter removal was individualized according to clinical recovery; cystography was performed only when urinary leakage was suspected.

### 2.4 Data collection and outcomes

Clinical, perioperative, pathological, and follow-up data were obtained from prospectively maintained institutional databases and verified against electronic medical records. Recorded variables included demographics, comorbidity, tumor characteristics, laboratory measurements, operative characteristics including lymph-node dissection, recovery outcomes, pathological findings, recurrence, and survival status.

Distal ureter and bladder cuff identification time was the sole primary endpoint and was measured retrospectively from archived full-length operative videos using the video timestamps. The original measurements were performed by a urologist investigator. The interval began when dedicated pelvic dissection of the distal ureter commenced at the level of the iliac vessel crossing and ended when the ureterovesical junction had been clearly identified and the planned circumferential bladder cuff margin had been delineated, before cystotomy or bladder cuff incision. The same anatomical boundaries were applied in both groups, and activation of near-infrared imaging did not define either boundary. The corresponding time entry in the operative record was used as a cross-check. For independent verification, a second urologist who was not involved in the original timing assessment reviewed all 82 operative videos using the same anatomical definitions without access to the original measurements or statistical results. Representative annotated examples demonstrating the application of these anatomical start and stop criteria are provided in **Supplementary Videos 1**, **2**.

Injection-specific adverse events were not captured in predefined database fields. Available perioperative records were retrospectively reviewed for documentation of new gross hematuria attributed to injection, unintended bladder injury during injection, symptomatic urinary tract infection requiring treatment, injection-site bleeding requiring intervention, clinically apparent unintended extravasation beyond the intended submucosal injection field, and allergic reaction. Technical failure was defined as failure to obtain a usable fluorescence landmark at the ureterovesical junction and was assessed from archived operative videos.

Total operative time, estimated blood loss, perioperative morbidity, conversion, and pathological margin status were evaluated as exploratory perioperative or safety outcomes. Drain duration, urethral catheter duration, and postoperative length of stay were collected as descriptive postoperative care-process measures rather than treatment-effect endpoints because their timing was determined by individualized clinical decisions and center-specific postoperative pathways. Intravesical recurrence-free survival (IVRFS), recurrence-free survival (RFS), and overall survival (OS) were prespecified as exploratory oncological outcomes. IVRFS was measured from T_0_ to first intravesical recurrence. RFS was measured from T_0_ to first extravesical recurrence or death, whichever occurred first. OS was measured from T_0_ to death from any cause. Patients without an event were censored at the last recorded follow-up.

### 2.5 Statistical analysis

Analyses were performed in R 4.4.1 (R Foundation for Statistical Computing, Vienna, Austria). Continuous variables were summarized as mean (standard deviation) or median (interquartile range). For inferential comparisons, Student’s t test or the Wilcoxon rank-sum test was used as appropriate. Drain duration, urethral catheter duration, and postoperative length of stay were summarized descriptively without between-group hypothesis testing. Unordered categorical variables were compared with chi-square or Fisher’s exact tests; rank-based tests were used for ordered categories. Analyses used available observations: catheterization time was recorded for 79 patients, and the other reported outcomes for all 82. Interobserver reliability of video-derived identification time was assessed using a two-way mixed-effects, single-measure, absolute-agreement intraclass correlation coefficient (ICC), with absolute between-reviewer differences summarized as median and interquartile range.

Absolute standardized mean differences (SMDs) were calculated from the unweighted patient-level data to describe baseline covariate balance. For continuous variables, the difference in group means was divided by the square root of the average of the group-specific variances; continuous-variable SMDs were based on means and variances regardless of the descriptive summary format. For binary variables, the difference in proportions was standardized using the corresponding binomial variances. For categorical variables with more than two levels, a generalized SMD based on the Mahalanobis distance between the group-specific vectors of category proportions was used. SMDs are reported as absolute values, with values < 0.10 conventionally indicating negligible imbalance.

IVRFS, RFS, and OS were summarized with Kaplan-Meier curves and compared by the log-rank test; univariable Cox models provided exploratory hazard ratios and 95% confidence intervals. Identification time was analyzed using log-linear regression, with the treatment-group coefficient exponentiated as a geometric mean ratio. Given the cohort size, the primary parsimonious model adjusted for age, body mass index, tumor location, center, and surgery period. An expanded sensitivity model additionally included ASA score, tumor size, and grade. Advanced pathological T stage was excluded from these adjustment sets because it was determined postoperatively and was added only in a sensitivity analysis. HC3 heteroskedasticity-robust confidence intervals, 2,000 patient-level bootstrap resamples, and leave-one-out analyses were used to assess robustness. Identification time was also examined within center and calendar-period strata. Effect modification by calendar period was examined using a group-by-period interaction term with HC3 robust standard errors. To assess surgeon-related confounding, a sensitivity model added operating surgeon as a fixed categorical covariate to the primary adjustment set, and effect modification was examined using a group-by-surgeon interaction with HC3 robust standard errors. For total operative time, sensitivity analyses first excluded patients undergoing lymph-node dissection or nodal sampling. Non-target operative time was then derived by subtracting distal ureter/bladder cuff identification time from both groups and ICG injection time from the ICG group. Tests were two-sided with p < 0.05 as the nominal significance threshold. Other perioperative and oncological findings were interpreted as exploratory, without adjustment for multiple comparisons.

## 3 Results

### 3.1 Baseline characteristics

The final cohort comprised 43 patients treated with ICG-guided RA-RNU and 39 treated without ICG. Conventional P values identified a difference only in age (median 77.0 vs 71.0 years, p = 0.028). Absolute SMDs were small to moderate for most baseline characteristics (0.058–0.217), with larger imbalances for pathological T stage (0.568) and age (0.442). The more prominent residual imbalances were addressed in the adjusted and sensitivity analyses (**Table 1**).

**Table 1:** Baseline characteristics.

Variable	ICG	non-ICG	P value	SMD
Sex			0.375	0.217
Male	20 (46.5%)	14 (35.9%)		
Female	23 (53.5%)	25 (64.1%)		
Age	77.00 [65.50, 85.50]	71.00 [64.00, 79.00]	**0.028**	0.442
BMI	23.74 [22.12, 25.91]	24.09 [22.46, 25.59]	0.831	0.114
Side			0.515	0.152
Left	22 (51.2%)	17 (43.6%)		
Right	21 (48.8%)	22 (56.4%)		
Location			0.499	0.179
Pelvis	28 (65.1%)	22 (56.4%)		
Upper ureter	15 (34.9%)	17 (43.6%)		
Grade			1.000	0.058
Low	4 (9.3%)	3 (7.7%)		
High	39 (90.7%)	36 (92.3%)		
ASA score			0.643	0.136
2	27 (62.8%)	27 (69.2%)		
3	16 (37.2%)	12 (30.8%)		
Tumor size (mm)	26.00 [16.00, 40.00]	25.00 [19.00, 40.00]	0.683	0.078
TNM stage			0.194	0.568
pTa	4 (9.3%)	1 (2.6%)		
pTis	1 (2.3%)	3 (7.7%)		
pT1	11 (25.6%)	16 (41.0%)		
pT2	8 (18.6%)	3 (7.7%)		
pT3	19 (44.2%)	16 (41.0%)		
Hemoglobin	120.19 ± 20.06	122.79 ± 17.13	0.593	0.140
ALB	37.91 ± 4.35	38.53 ± 4.05	0.469	0.148

ICG, indocyanine green; ALB, albumin; ASA, American Society of Anesthesiologists; BMI, body mass index; SMD, standardized mean difference. Values are n (%), median [interquartile range], or mean ± standard deviation. Absolute SMDs are reported; for categorical variables with more than two levels, generalized SMDs were calculated. Bold values indicate statistical significance (p < 0.05).

### 3.2 Perioperative outcomes

Median distal ureter and bladder cuff identification time was 9.0 min with ICG guidance and 19.3 min without ICG (p < 0.001); ICG injection took a median of 5.3 min (IQR 4.6–6.1). In the primary parsimonious model, ICG guidance was associated with shorter identification time (geometric mean ratio, 0.489; HC3 95% CI, 0.449–0.533; p < 0.001). The estimate was similar in the expanded measured-covariate model (0.489; 95% CI, 0.444–0.537) and when advanced pathological T stage was added only as a sensitivity variable (0.489; 95% CI, 0.446–0.537). Bootstrap and leave-one-out analyses yielded consistent results (**Supplementary Tables 2**, **5**). Both surgeons contributed ICG and non-ICG cases (Surgeon 1, 15 and 19; Surgeon 2, 28 and 20, respectively; Fisher’s exact p = 0.263). Adding operating surgeon to the primary model did not materially change the association (geometric mean ratio, 0.488; HC3 95% CI, 0.446–0.534; p < 0.001), and the group-by-surgeon interaction was not significant (HC3 p = 0.762). Identification-time medians were similar across calendar periods within each group (ICG, 8.8 vs 9.1 min; non-ICG, 19.2 vs 19.3 min), with no group-by-period interaction (HC3 p = 0.947; **Supplementary Tables 2**, **5**).

Among the 43 patients who underwent submucosal ICG marking, no new gross hematuria attributed to injection, unintended bladder injury during injection, symptomatic urinary tract infection requiring treatment, injection-site bleeding requiring intervention, clinically apparent unintended extravasation, or allergic reaction was documented during the index hospitalization. A usable fluorescence landmark was identified in all 43 archived operative videos, and no technical failure was observed.

Other perioperative outcomes are summarized in **Table 2**. Median total operative time was 170 min in the ICG group and 230 min in the non-ICG group (p < 0.001), but this was an unadjusted exploratory comparison that included other components of the operation. Estimated blood-loss distributions differed between groups (interquartile range, 50–100 mL vs 100–200 mL in the ICG and non-ICG groups, respectively; Wilcoxon rank-sum p < 0.001). Drain duration, urethral catheter duration, and postoperative length of stay are reported descriptively in **Table 2** without between-group hypothesis testing. Pain score, ICU admission, bowel recovery, postoperative hemoglobin and albumin, and Clavien-Dindo grade did not differ statistically.

**Table 2:** Perioperative outcomes and postoperative care measures.

Variable	ICG (n = 43)	non-ICG (n = 39)	P value
Primary outcome			
Distal ureter and bladder cuff identification time (min)	9.00 [8.45, 9.45]	19.30 [18.75, 20.25]	<0.001
Exploratory perioperative outcomes			
ICG injection time (min)	5.30 [4.60, 6.10]	Not applicable	—
Total operative time (min)	170.00 [132.50, 227.50]	230.00 [192.50, 267.50]	<0.001
Estimated blood loss (mL)	100.00 [50.00, 100.00]	100.00 [100.00, 200.00]	<0.001
VAPS at 24 h	4.00 [3.00, 6.50]	4.00 [2.00, 7.00]	0.779
Open conversion	0 (0.0%)	0 (0.0%)	—
ICU admission	8 (18.6%)	8 (20.5%)	1.000
Positive surgical margin	0 (0.0%)	2 (5.1%)	0.223
Time to bowel function recovery (d)	3.00 [2.00, 3.00]	2.00 [1.00, 3.00]	0.387
Clavien–Dindo grade			0.297
0	36 (83.7%)	29 (74.4%)	
1	2 (4.7%)	0 (0.0%)	
2	4 (9.3%)	8 (20.5%)	
3	0 (0.0%)	1 (2.6%)	
4	1 (2.3%)	1 (2.6%)	
Postoperative hemoglobin (g/L)	107.51 ± 18.52	109.67 ± 14.07	0.707
Postoperative albumin (g/L)	33.20 [29.65, 34.20]	33.20 [31.30, 34.75]	0.419
Descriptive postoperative care measures			
Drain duration (d)	5.00 [4.00, 5.00]	8.00 [6.00, 11.00]	—
Urethral catheter duration (d)	6.00 [3.00, 8.00]	8.00 [7.00, 10.25]	—
Postoperative hospital stay (d)	6.00 [5.00, 8.00]	11.00 [11.00, 12.00]	—

Values are median [IQR], mean ± SD, or n (%). Catheter duration was available for 43 ICG and 36 non-ICG patients. ICG, indocyanine green; ICU, intensive care unit; IQR, interquartile range; SD, standard deviation; VAPS, visual analog pain scale.

Lymph-node procedures were similarly distributed between groups (7/43 [16.3%] vs 8/39 [20.5%]; p = 0.776). After excluding patients who underwent lymph-node dissection or nodal sampling, the median difference in total operative time decreased from 60 to 19 min. After subtracting identification time from both groups and ICG injection time from the ICG group, median non-target operative time was 176.45 versus 191.30 min, with a between-group difference of −14.85 min (95% bootstrap CI, −56.75 to 21.90; p = 0.069; **Supplementary Table 4**). Open conversion did not occur. Positive surgical margins occurred in 2/39 non-ICG patients and 0/43 ICG patients (p = 0.223); both were peripelvic soft-tissue margins in patients with pT3 renal-pelvic tumors (**Table 2**). Adjusted and stratified analyses are reported in **Supplementary Table 2**, with additional robustness checks in **Supplementary Table 5** and sensitivity analyses of total operative time in **Supplementary Table 4**.

### 3.3 Exploratory oncological outcomes

Oncological outcomes were analyzed on an exploratory basis. All 82 patients received postoperative intravesical pirarubicin according to the protocol described above. Median follow-up was 25.3 months in the ICG group and 22.3 months in the non-ICG group (p = 0.613). Intravesical recurrence occurred in 1/43 and 2/39 patients, respectively; extravesical recurrence occurred in 3/43 and 8/39. The small number of events and limited follow-up precluded conclusions regarding oncological safety or any effect of ICG marking on recurrence. Both margin-positive patients received four cycles of adjuvant gemcitabine–cisplatin. One remained recurrence-free at 25.03 months; the other developed a pTa low-grade intravesical recurrence at 35.26 months, which was treated with TURBT and immediate intravesical mitomycin C, with no further recurrence through 44.83 months.

In exploratory Kaplan–Meier analyses, log-rank p values were 0.401, 0.113, and 0.254 for IVRFS, RFS, and OS, respectively (**Supplementary Figure S1**). Univariable Cox estimates are reported descriptively in **Supplementary Table 3**.

## 4 Discussion

In this multicenter cohort, cystoscopic submucosal ICG marking was associated with shorter distal ureter and bladder cuff identification time during RA-RNU. The association remained consistent across the parsimonious primary model, expanded sensitivity analyses, and the two calendar periods examined. A submucosal depot marks the ureterovesical junction before pelvic dissection, providing a stable fluorescent reference where pelvic depth, perivesical fat, and the absence of tactile feedback can obscure the distal ureter.

During robotic cuff excision, the lower ureter can be difficult to locate in a deep pelvic field, particularly when perivesical tissue obscures the usual planes (21). By outlining the ureterovesical junction before cystotomy, submucosal ICG provides a visible starting point for defining the cuff margin. Although the fluorescence landmark may assist localization, its effects on the extent of dissection, tissue traction, and postoperative recovery were not evaluated. The robotic platform may also facilitate precise distal ureter and bladder cuff dissection; recent comparative data reported lower intravesical recurrence after robotic RNU in an exploratory pT3 subgroup, although overall oncological outcomes were similar and this observation requires further validation (22).

Route of administration determines where fluorescence appears. Near-infrared ICG imaging is established in robotic urology (23). For ureteral mapping in UTUC, the aim is to make the distal ureter visible without pressurizing the tumor-bearing lumen. Retrograde instillation provides intraluminal fluorescence, and retrospective UTUC series have linked luminal manipulation with subsequent bladder recurrence (17–19). In this technique, a low-pressure submucosal depot around the ureteral orifice produces an extraluminal signal at the bladder cuff. Because injection in patients with distal ureteral tumors would place the needle close to tumor-bearing tissue, these tumors were not included in the present cohort, consistent with established oncological principles for RNU (24, 25). This study did not compare ICG administration routes. In both groups, the ureter was clipped before distal mobilization and again before bladder cuff excision, with bladder decompression maintained before urinary tract entry, consistent with established oncological principles for RNU (24, 25).

The principal finding was a 10.3-min difference in median distal ureter and bladder cuff identification time (9.0 vs 19.3 min). ICG injection itself required a median of 5.3 min, and the crude 60-min difference in total operative time was too large to be attributed to the localization step. After excluding patients undergoing lymph-node procedures, the median total operative-time difference decreased to 19 min. After identification and injection times were subtracted, the remaining difference in non-target operative time was not statistically significant (−14.85 min; 95% bootstrap CI, −56.75 to 21.90; p = 0.069; **Supplementary Table 4**). We therefore interpret faster distal ureter and bladder cuff identification as the principal finding.

Oncological outcomes were exploratory, and the small number of events precluded comparative inference. The numerical differences in recurrence should not be interpreted as evidence that ICG marking reduces recurrence. The two positive margins in the non-ICG group involved peripelvic soft tissue in patients with pT3 renal-pelvic tumors; neither involved the distal ureter or bladder cuff. The relatively short follow-up further precludes conclusions regarding oncological safety.

Several limitations warrant attention. First, treatment allocation followed local practice rather than random assignment. Choice of ICG may therefore have reflected case complexity, center-specific practice, or surgeon preference. Residual baseline imbalance was most pronounced for age and pathological T stage. Age was included in the primary adjusted model, whereas pathological T stage was assessed only in a sensitivity analysis because it was determined postoperatively. Despite these analyses, residual and unmeasured confounding cannot be excluded. Second, the cohort comprised only 82 patients, including 43 ICG-guided procedures, limiting precision for exploratory outcomes and precluding meaningful subgroup analyses. Injection-specific adverse events were assessed retrospectively, and patient-level injected volumes were not consistently documented; consequently, uncommon or delayed injection-related events cannot be excluded, and dose-specific safety could not be assessed. Third, identification time was assessed retrospectively from operative videos using predefined anatomical landmarks. Although interobserver agreement was excellent, treatment-group blinding was not feasible because fluorescence was visible during ICG-guided procedures, and some observer judgment remained in identifying the start and stop boundaries. Fourth, procedures were performed or supervised by two high-volume robotic surgeons. The estimate was unchanged after adjustment for operating surgeon, and neither the group-by-surgeon nor group-by-period interaction was significant. However, exact center-specific ICG introduction dates and chronological case numbers could not be reconstructed consistently; calendar period therefore remained only a coarse proxy for cumulative experience and temporal changes in operative practice. Potential center-specific differences in postoperative pathways also preclude causal interpretation of postoperative length of stay and the timing of drain and urethral catheter removal. In addition, distal ureteral tumors were not represented in the analytic cohort; therefore, these findings should not be extrapolated to tumors involving the intramural ureter or ureterovesical junction. Finally, median follow-up was 25.3 and 22.3 months in the ICG and non-ICG groups, respectively, and oncological events were uncommon, precluding conclusions regarding longer-term oncological safety or any effect of ICG marking on recurrence. Prospective multicenter studies should incorporate standardized ICG dosing, prespecified injection-specific safety surveillance, video-based adjudication, standardized recovery pathways, and longer follow-up for oncological outcomes.

## 5 Conclusions

Cystoscopic submucosal ICG marking was associated with faster identification of the distal ureter and bladder cuff during RA-RNU. By providing a readily visible extraluminal landmark at the ureterovesical junction, this approach may facilitate localization during the lower-tract phase of the procedure while avoiding intraluminal ICG instillation. Prospective studies should standardize the administered ICG volume and prospectively evaluate injection-specific safety, reproducibility, and longer-term oncological outcomes.

## Data Availability

Because the dataset contains sensitive clinical information, it cannot be deposited in a public repository. Requests for a de-identified analytic dataset should be directed to the corresponding author; release requires institutional authorization and a data-use agreement.

## Supplementary material

Supplementary Video 1 Annotated representative operative video demonstrating the prespecified measurement of distal ureter and bladder cuff identification time during RA-RNU. The measured source-video interval is 00:13:29–00:20:52 (7 min 23 s); the submitted excerpt spans 00:13:24–00:20:57.

Supplementary Video 2 Annotated representative operative video demonstrating the prespecified measurement of distal ureter and bladder cuff identification time during RA-RNU. The measured source-video interval is 00:14:11–00:24:36 (10 min 25 s); the submitted excerpt spans 00:14:06–00:24:41.
