# Supplementary material for: Cystoscopic submucosal indocyanine green marking in robot-assisted radical nephroureterectomy: a multicenter cohort study
Source: Front Oncol. 2026 Sep 28;16:1957208. doi: 10.3389/fonc.2026.1957208 (PMC13654764; doi:10.3389/fonc.2026.1957208)
Supplement: Supplementary file 1 [file DataSheet1.pdf]

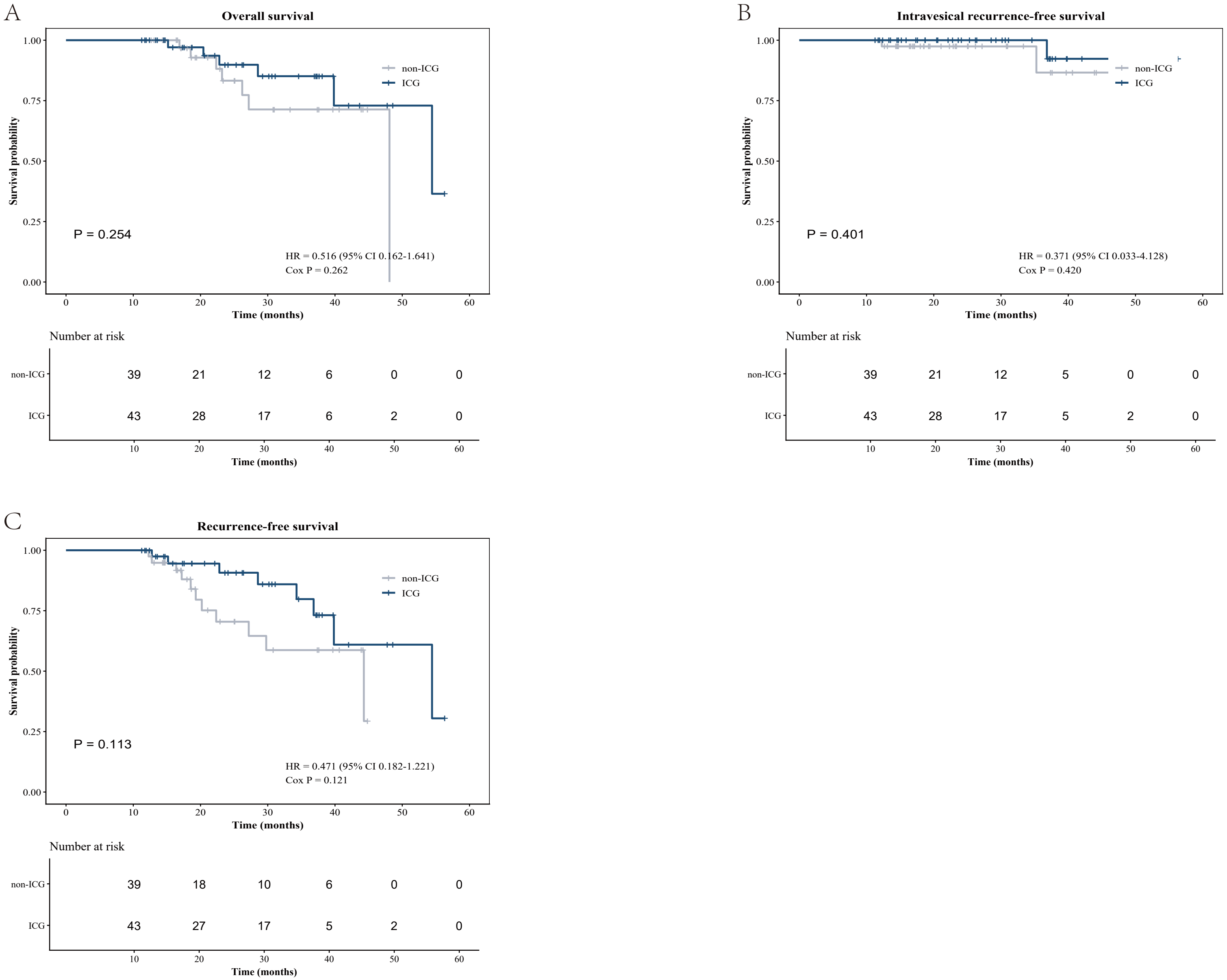

Supplementary Figure S1. Exploratory oncological safety surveillance according to treatment group. **(A)** Overall survival. **(B)** Intravesical recurrence-free survival. **(C)** Recurrence-free survival. Events were uncommon (intravesical recurrence, 1/43 vs 2/39; extravesical recurrence, 3/43 vs 8/39 in the ICG and non-ICG groups, respectively). Numbers at risk are shown below each curve. Panels display log-rank p values and exploratory hazard ratios. Abbreviations: CI, confidence interval; HR, hazard ratio; ICG, indocyanine green; IVRFS, intravesical recurrence-free survival; OS, overall survival; RFS, recurrence-free survival.
